# A conservation checklist of the amphibians and reptiles of Sonora, Mexico, with updated species lists

**DOI:** 10.3897/zookeys.829.32146

**Published:** 2019-03-11

**Authors:** Julio A. Lemos-Espinal, Geoffrey R. Smith, James C. Rorabaugh

**Affiliations:** 1 Laboratorio de Ecología-UBIPRO, FES Iztacala UNAM, Avenida los Barrios 1, Los Reyes Iztacala, Tlalnepantla, edo. de Mexico, 54090, Mexico Universidad Nacional Autónoma de México Tlalnepantla Mexico; 2 Department of Biology, Denison University, Granville, Ohio 43023, USA Denison University Granville United States of America; 3 P.O. Box 31, Saint David, Arizona 85630, USA Unaffilaited Phoenix United States of America

**Keywords:** United States-Mexico border states, ecoregions, herpetofauna, IUCN Red List, shared species

## Abstract

Sonora has a rich natural diversity, including reptiles and amphibians. Sonora’s location on the United States-Mexico border creates some unique conservation challenges for its wildlife. We compiled a list of the amphibian and reptile species currently known for Sonora, summarized the conservation status of these species, and compared our list of species with known species lists for adjacent states. The herpetofauna of Sonora comprises 200 species of amphibians and reptiles (38 amphibians and 162 reptiles). Overall, Sonora shares the most species with Chihuahua, Sinaloa, and Arizona. Approximately 11% of the amphibian and reptile species are IUCN listed, but 35.5% are placed in a protected category by SEMARNAT, and 32.6% are categorized as high risk by the Environmental Vulnerability Score.

## Introduction

**Figure 1.**
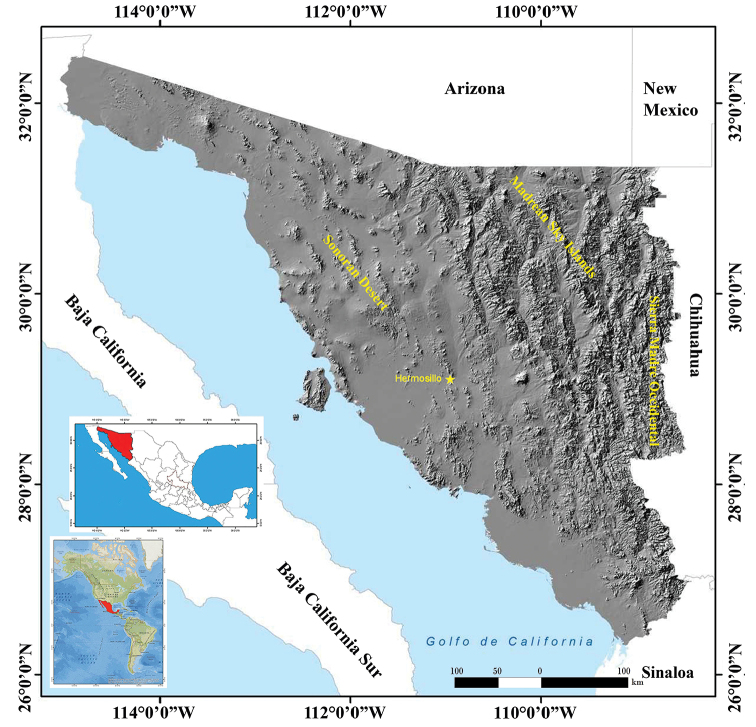
Topographical map of the state of Sonora, Mexico (INEGI 2009). Map of America modified from http://www.gifex.com/fullsize/2009-09-17-3/Mapa-de-Amrica.html; Map of Mexico with the state of Sonora in red modified from Comisión Nacional para el Conocimiento y Uso de la Biodiversidad (2008).

**Figure 2.**
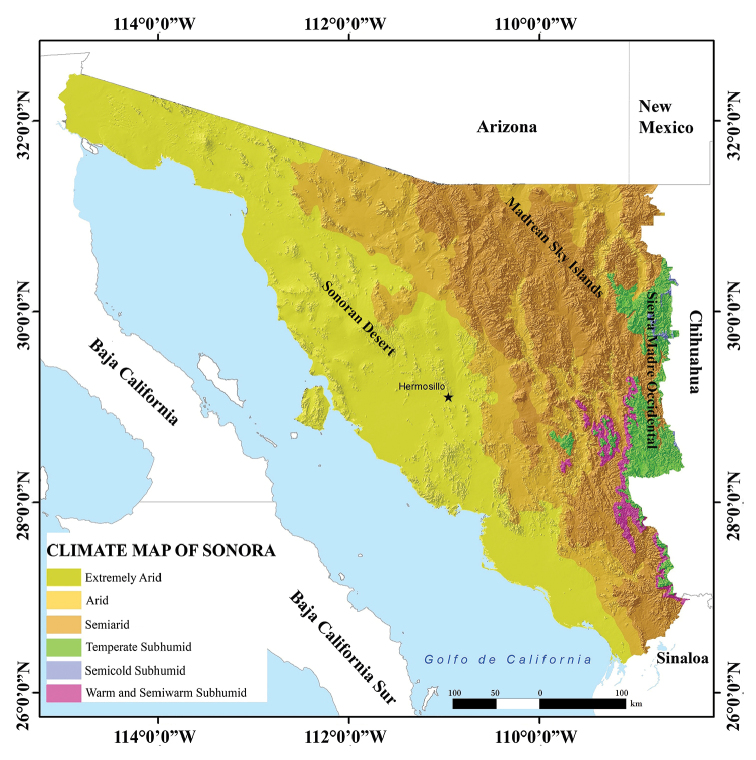
Climate map of the state of Sonora, Mexico (modified from García – CONABIO 1998).

Sonora is a state that, due to its geographic location near the U.S. states of Arizona and California and the extraordinary natural diversity those states host, has attracted the attention of specialists and amateurs in the study of its flora and fauna. Therefore, Sonora’s biodiversity is perhaps the best known among the states of northern Mexico. Sonora’s varied topography and climate (Figs 1, 2); with altitudes ranging from sea level to 2,625 m, broad plains in the west, high mountains in the east, islands in the Gulf of California, and more than 1,200 km of coastline; have resulted in high levels of biodiversity. Sonora is also home to relatively unique habitats, such as the peat moss habitat found in the Ciénega de Camilo in eastern Sonora (Van Devender et al. 2003), and the spring-fed wetlands or ciénegas of the Apache Highlands of Arizona and Sonora (Minckley et al. 2013). Sonora is also part of the main “hot spot” of tropical dry forests; however, climate change is likely to result in degradation of these forests as is deforestation and increased clearing for agriculture (Prieto-Torres et al. 2016).

Given its physiographic and topographic diversity, Sonora is home to high levels of biodiversity, including its herpetofauna (see Lemos-Espinal and Rorabaugh 2015). In particular, Sonora has several areas that are important with respect to herpetofaunal diversity. The desert shrubland in Sonora supports a high diversity of lizards due to the abundance of microhabitats it provides (García and Whalen 2003). Sonora is the location of the southern range limits of several arid adapted reptiles and amphibians (Bezy et al. 2017), but also the location of the northern limits of Neotropical species (Lavín-Murcio and Lazcano 2010). The Northern Jaguar Reserve in Sonora houses a mixture of amphibians and reptiles from a variety of macrohabitat and biogeographic regions (Rorabaugh et al. 2011). The Pacific Lowlands, including areas of Sonora, are one of the more critical areas of endemism for reptiles and amphibians in Mexico (Johnson et al. 2017).

The location of Sonora along the United States-Mexico border creates some unique issues for the conservation of its wildlife. Environmental quality and ecosystem services on the Mexican side of the Sonora-Arizona border are declining (Norman et al. 2012b). One challenge confronting Sonora’s environment is human population growth and urbanization. This is particularly important along the U.S.-Mexico border as the human population of Nogales, Mexico is rapidly increasing (Norman et al. 2009, 2012a), which is consistent with a general trend in the border region (Anderson 2003). There has also been an increase in economic growth in Sonora, especially agriculture and ranching (Magaña and Conde 2000). Grazing by cattle can result in the loss of important native vegetation and alteration of Sonoran habitats (Morales-Romero et al. 2012). Such development will potentially result in major losses in habitats, such as riparian woodlands and semi-desert grasslands in the region (Villarreal et al. 2013). Other conservation concerns include non-native species (Bogan et al. 2014, Drake et al. 2017), habitat fragmentation that reduces demographic and genetic connectivity (e.g., across the international border due to construction of walls and other infrastructure on the U.S. side; Peters et al. 2018), and climate change resulting in changes in temperature and precipitation (Stahlschmidt et al. 2011, Flesch et al. 2017, Griffis-Kyle et al. 2018).

Another challenge to Sonora’s environment is related to water usage. Watersheds in the region are subject to increasing urbanization, ranching, and losses due to irrigation (Steiner et al. 2000). Increased human populations in Sonora will also drain freshwater for domestic uses and for power generation (Magaña and Conde 2000, Scott et al. 2012). Also, some freshwater systems in Sonora are subject to salinization due to intrusion of saltwater into freshwater aquifers as a result of pumping of water from the aquifers for human use (Contreras-B. and Lozano-V. 1994, Halvorson et al. 2003). Climate change is also likely to increase the strain on freshwater aquifers in Sonora (Scott et al. 2012) and the region encompassing the US-Mexico border areas (Ye and Grimm 2013).

The factors mentioned above are likely to affect several taxonomic groups, but the herpetofauna is a group of particular concern. Rorabaugh (2008) found that 40% of the Sonoran herpetofauna were given some conservation status by the Mexican government (SEMARNAT) or the IUCN Red List. Although there have been several recent works that report lists of species of reptiles and amphibians in Sonora (Rorabaugh 2008, Enderson et al. 2009, 2010, Lemos-Espinal and Smith 2009, Lemos-Espinal and Rorabaugh 2015, Lemos-Espinal et al. 2015, Rorabaugh and Lemos-Espinal 2016), species additions and accelerating taxonomic changes merit a new analysis of the current list for Sonora, especially with respect to the conservation status of the species listed. Here, we report the list of species currently known for the state of Sonora, focusing on the conservation status reported for each species, analyzing it by taxonomic groups and ecoregions, and comparing our list of species with known lists for adjacent states.

## Methods

We only included species in the checklist for which we could confirm the record in Sonora, either by direct observation or through documented museum records or vouchers. We follow Frost (2018) or AmphibiaWeb (2018) for amphibian names and Uetz and Hošek (2018) for reptile names (for a summary of recent taxonomic changes see Table 1). We compiled the list of amphibians and reptiles of the state of Sonora from the following sources: (1) our own field work; (2) specimens from the Amphibians and Reptiles collection of the University of Arizona; (3) specimens from the Laboratorio de Ecología – UBIPRO (LEUBIPRO) collections; (4) a thorough examination of the available literature on amphibians and reptiles in the state; (5) amphibian and reptile records for the state of Sonora in VertNet.org; and (6) databases from the Comisión Nacional para el Conocimiento y Uso de la Biodiversidad (CONABIO, or National Commission for the Understanding and Use of Biodiversity) (see Appendix 1).

**Table 1.** Recent taxonomic changes for the herpetofauna of Sonora.

Taxon	Explanation
* Rhinella horribilis *	Acevedo et al. (2016) demonstrated that there were two separate evolutionary lineages within *Rhinellamarina* representing two distinct species: *R.marina* for the eastern populations, and *R.horribilis* for the western populations.
* Dryophytes *	We use *Dryophytes* based on Duellman et al. (2016).
* Rana *	Frost et al. (2006) recommended the use of the name *Lithobates* for North American *Rana*. However, we use *Rana* because Yuan et al. (2016) recently returned all *Lithobates* to *Rana*, based on a phylogenetic analysis of six nuclear and three mitochondrial loci sampled from most species of *Rana*, the lack of any diagnostic morphological characters for the genera recognized by Frost et al. (2006), and the clear monophyly of a larger group that include these genera.
* Isthmura sierraoccidentalis *	Originally *Isthmurasierraoccidentalis* was described as a subspecies of *Pseudoeuryceabelli* by Lowe et al. (1968), recently it was elevated to full species status by Rovito et al. (2015).
* Aspidoscelis *	Tucker et al. (2016), based on Steyskal (1971), explained and justified why the genus name *Aspidoscelis* should be treated as masculine, thus we use the appropriate masculine species names.
* Boa *	Card et al. (2016) recently recognized the *Boa* populations from the slopes of the Mexican Pacific as *Boasigma*, which we follow.
* Chionactis annulata *	Wood et al. (2014) raised *Chionactisoccipitalisannulata* to full species status (*C.annulata*).
*Chionactis*, *Chilomeniscus*, and *Sonora*	Cox et al. (2018) concluded that *Sonora* is paraphyletic with respect to *Chilomeniscus* and *Chionactis* and found additional evidence to suggest synonomizing *Chionactis* and *Chilomeniscus* with *Sonora*. However, due to the long history of the use of the names of these three genera, we retain the use of the three genera to reduce confusion. In addition, other interpretations of the work of Cox et al. (2018) leave the current arrangement in place instead of synonymizing them (A Holycross and D Wood pers. comm.).
* Lampropeltis *	Based on the work of Krysko et al. (2017) the state of Sonora hosts three species of the *Lampropeltisgetula* complex: *Lampropeltiscaliforniae* along most of the border with Arizona; *Lampropeltissplendida* in the northeastern corner of the state, in the region where Arizona, New Mexico, Chihuahua and Sonora converge; and *Lampropeltisnigrita*, occupying most of the state of Sonora, including the islands of Tiburón and San Pedro Nolasco.
* Crotalus pyrrhus *	Meik et al. (2015) elevated *Crotalusmitchellipyrrhus* to full species status, so we report *C.pyrrhus* as occurring in Sonora.

**Figure 3.**
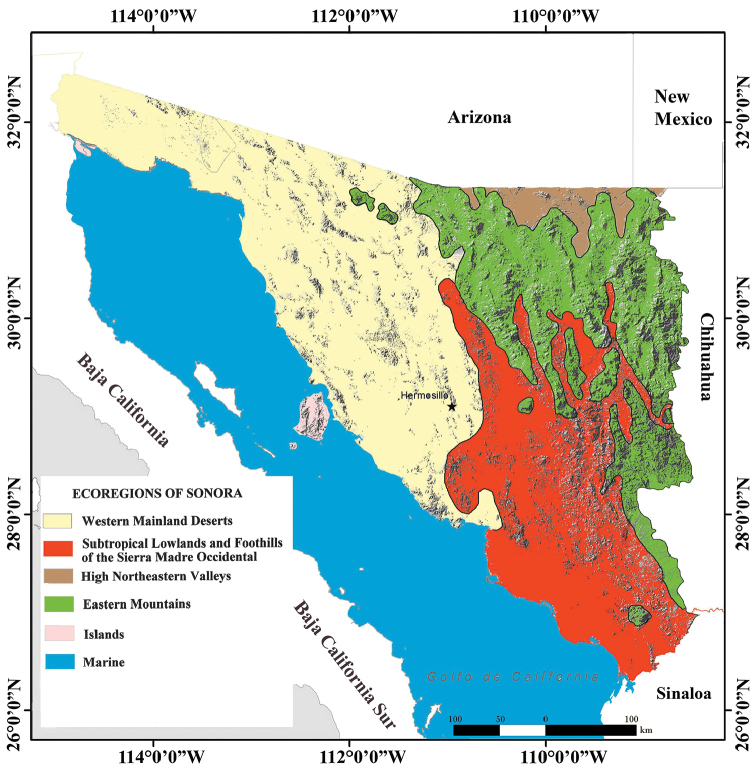
Map of the ecoregions of the state of Sonora, Mexico (created by J Rorabaugh using the base topographic map of INEGI 2009).

**Figure 4.**
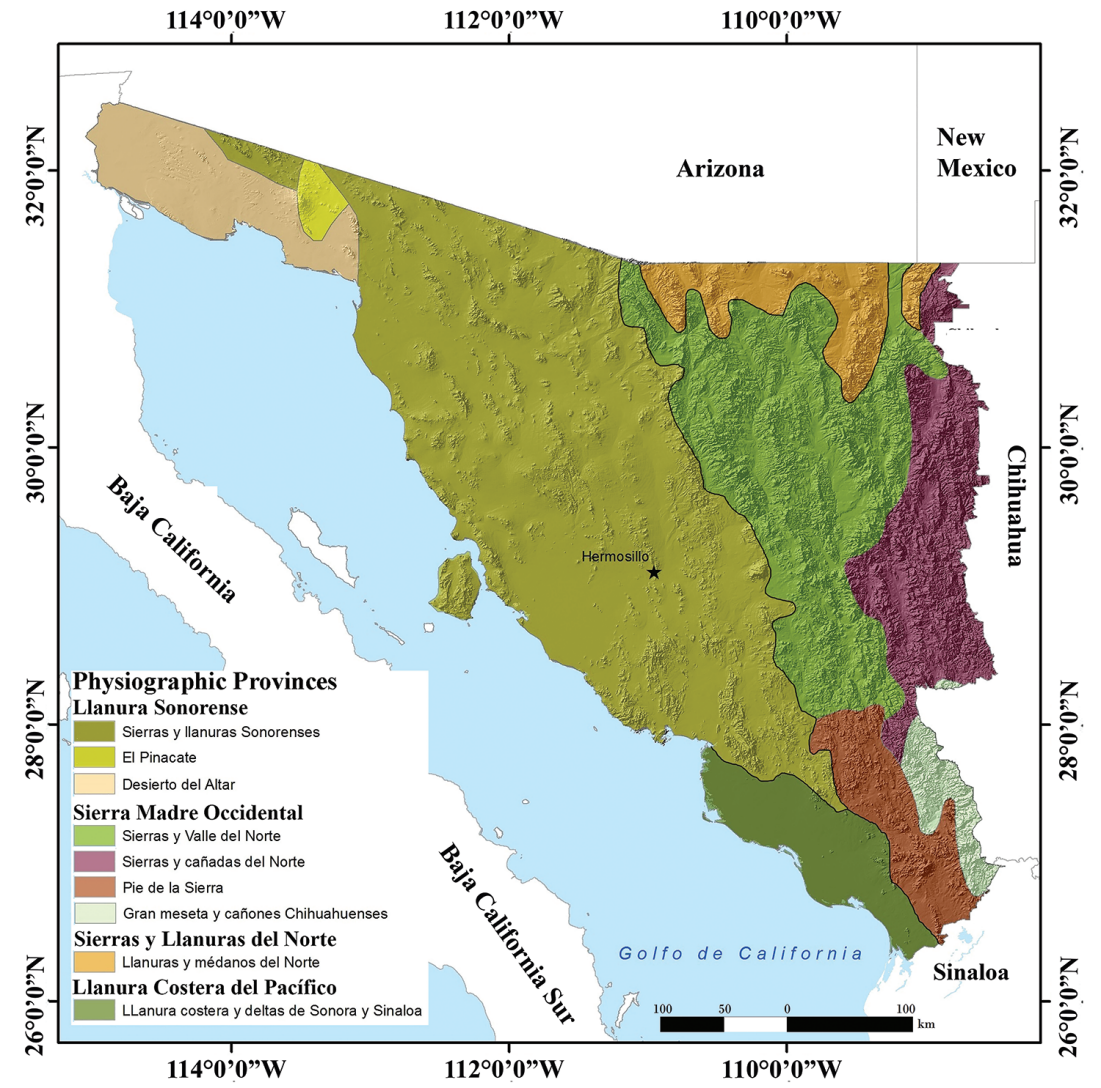
Topographical map with physiographic provinces of the state of Sonora, Mexico. Map modified from Cervantes-Zamora et al. (1990).

**Figure 5.**
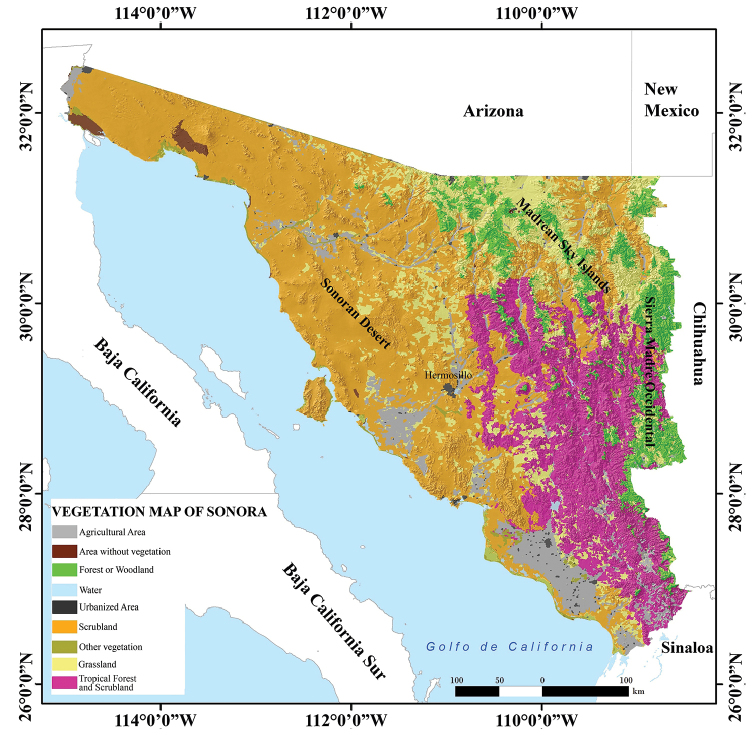
Vegetation type map of the state of Sonora, Mexico (modified from Dirección General de Geografía – INEGI 2005).

We recognize six herpetological ecoregions in Sonora (Eastern Mountains, High Northeastern Valleys, Western Mainland Deserts, Subtropical Lowlands and Foothills of the Sierra Madre Occidental, Islands, and Marine), each of which supports distinctive amphibian and reptile assemblages (Fig. 3). These ecoregions are further defined by geography, elevational range, topography, and vegetation communities (see Lemos-Espinal and Rorabaugh 2015; Lemos-Espinal et al. 2015; Rorabaugh and Lemos-Espinal 2016 for a description of these ecoregions). As a result, boundaries of ecoregions bear some resemblance to those of physiographic units (Fig. 4) and vegetation communities (Fig. 5).

We recorded the conservation status of each species based on 1) the IUCN Red List 2018-2; 2) Environmental Viability Scores from Wilson et al. (2013a, b); and 3) listing in SEMARNAT (2010). The number of overlapping species with the five neighboring states of Sonora was determined using recent state lists (Arizona, Brennan and Babb [2015]; Baja California, Hollingsworth et al. [2015]; Sinaloa, Enderson et al. [2009]; Chihuahua, Lemos-Espinal et al. [2017]; and New Mexico, Painter and Stuart [2015]). Lists were updated for Arizona (adding *Lampropeltiscaliforniae* [Blainville] and *L.nigrita* Zweifel & Norris, and substituting *Lampropeltissplendida* [Baird & Girard] for *L.getula* Linnaeus [Krysko et al. 2017]); Baja California (substituting *Lampropeltiscaliforniae* [Blainville] for *L.getula* Linnaeus [Krysko et al. 2017]); Sinaloa (adding *Crocodylusacutus* Cuvier [Natural History Museum of Los Angeles County. LACM Vertebrate Collection. Record ID: D411FDF6-C9FA-471B-BC83-B1FC044E54C3. Source: http://ipt.vertnet.org:8080/ipt/resource.do?r=lacm_verts [accessed on 2018-03-13]], *Leptodeirasplendida* Günther [Natural History Museum of Los Angeles County. LACM Vertebrate Collection. Record ID: 6CD2EBCD-71BA-426B-A9A2-9DF8FE3222B5. Source: http://ipt.vertnet.org:8080/ipt/resource.do?r=lacm_verts (accessed on 2018-03-13)], and *Gopherusevgoodei*, Edwards et al. 2016, and substituting *Lampropeltisnigrita* Zweifel & Norris for *L.getula* Linnaeus [Krysko et al. 2017]); Chihuahua (substituting *Sceloporuscowlesi* Lowe & Norris for *S.consobrinus* Baird & Girard [A Leaché, pers. comm., April 2017]); and New Mexico (adding *Lampropeltisholbrooki* Stejneger, and substituting *Lampropeltissplendida* [Baird & Girard] for *L.getula* Linnaeus [Krysko et al. 2017]).

We created species accumulation curves for the total herpetofauna, amphibians, and reptiles using the year of the first recorded observation for each species. Such species accumulation curves are likely to be reasonable estimates of the species richness of amphibians and reptiles (see Raxworthy et al. 2012).

## Results and discussion

Sonora hosts a total of 200 (seven of them introduced) species of amphibians and reptiles. This is an increase of four species from the list compiled by Rorabaugh and Lemos-Espinal (2016), and 13 species from the list compiled by Enderson et al. (2009). Thirty-eight are amphibians (35 anurans [two introduced], and three salamanders) and 162 reptiles (one crocodile, 69 lizards [three introduced], 75 snakes [one introduced], and 17 turtles [one introduced]) (Tables 2, 3). These represent 38 families: ten amphibians (eight anurans, one salamanders), and 28 reptiles (one crocodile, 12 lizards [one introduced], eight snakes [one introduced], and seven turtles [one introduced]). Sonora has 91 genera: 17 amphibians (15 anurans, two salamanders), and 74 reptiles (one crocodile, 22 lizards [one introduced], 40 snakes [one introduced], and eleven turtles [one introduced]). Twelve of the 193 native species are only found in islands in Sonora, those are: Isla San Esteban Spiny-tailed Iguana (*Ctenosauraconspicuosa*), Isla San Pedro Nolasco Spiny-tailed Iguana (*C.nolascensis*), Piebald Chuckwalla (*Sauromalusvarius*), Isla San Pedro Nolasco Lizard (*Utanolascensis*), Isla San Pedro Mártir Side-blotched Lizard (*U.palmeri*), Peninsular Leaf-toed Gecko (*Phyllodactylusnocticolus*), San Pedro Nolasco Gecko (*P.nolascoensis*), San Pedro Nolasco Whiptail (*Aspidoscelisbacatus*), San Esteban Whiptail (*A.estebanensis*), San Pedro Mártir Whiptail (*A.martyris*), Isla San Esteban Whipsnake (*Masticophisslevini*), and Isla San Esteban Black-tailed Rattlesnake (*Crotalusestebanensis*). Another seven are marine species: American Crocodile (*Crocodylusacutus*), Yellow-bellied Seasnake (*Hydrophisplaturus*), Loggerhead Sea Turtle (*Carettacaretta*), Green Sea Turtle (*Cheloniamydas*), Hawksbill Sea Turtle (*Eretmochelysimbricata*), Olive Ridley Sea Turtle (*Lepidochelysolivacea*), and Leatherback Sea Turtle (*Dermochelyscoriacea*). The introduced species are: Rio Grande Leopard Frog (*Ranaberlandieri*), American Bullfrog (*R.catesbeiana*), Common House Gecko (*Hemidactylusfrenatus*), Mediterranean House Gecko (*H.turcicus*), Spiny Chuckwalla (*Sauromalushispidus*), Brahminy Blindsnake (*Indotyphlopsbraminus*), and Spiny Softshell (*Apalonespinifera*).

**Figure 6.**
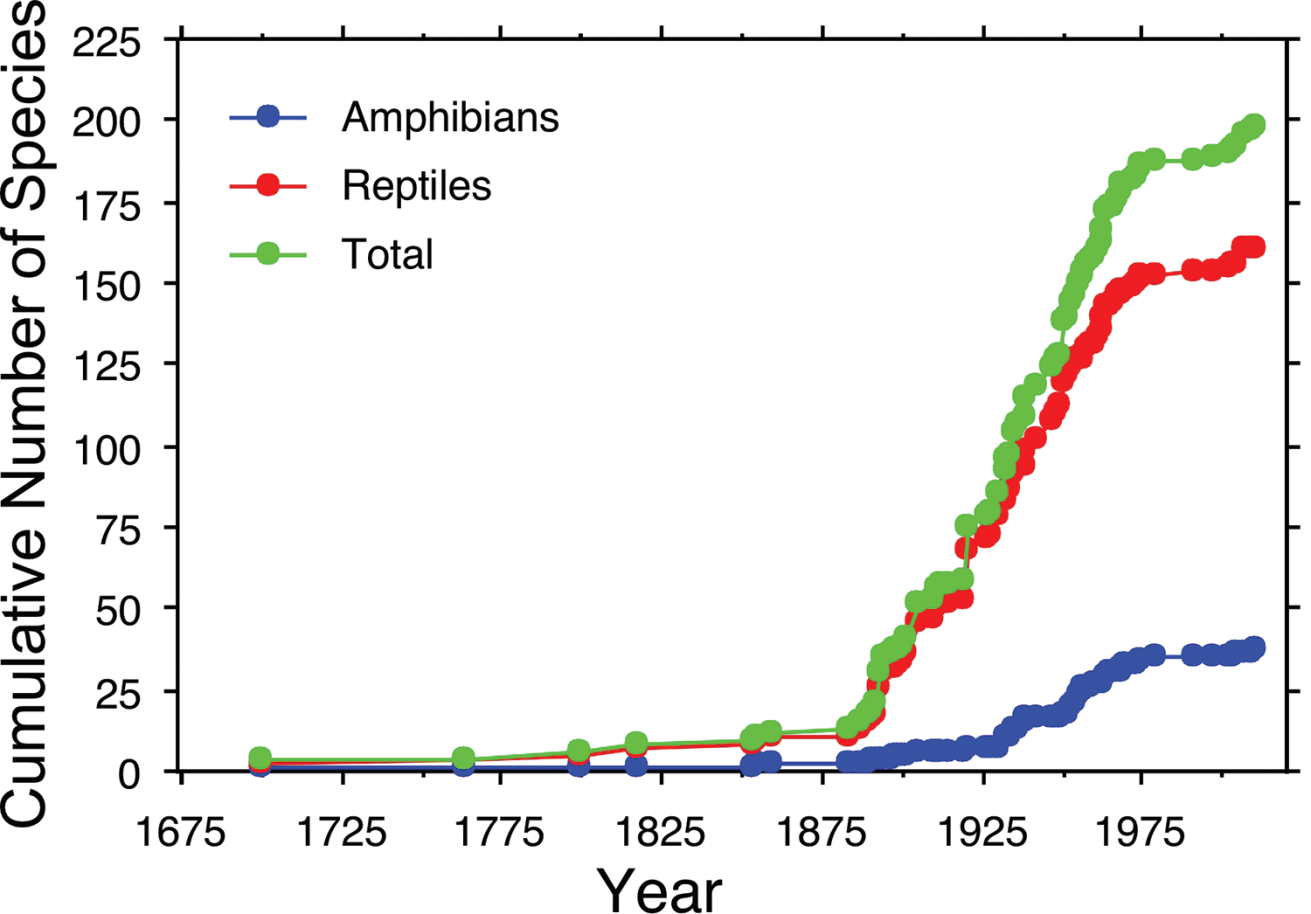
Species accumulation curves for the amphibians, reptiles, and total herpetofauna of Sonora, Mexico.

The species accumulation curves for all species, amphibians only, and reptiles only suggest that the current list of species likely underestimates the species richness for Sonora (Fig. 6). These curves show a rapid increase in species during the first half of the 20^th^ century with a steady, almost linear, increase in the number of species recorded in Sonora. Following a brief period of little additional accumulation of new species recorded in Sonora in the late 1900’s, there has been a recent increase in the number of species added to the Sonoran herpetofauna. This increase includes recent documentation of non-native species (*Apalonespinifera*, *Hemidactylusfrenatus*, and *H.turcicus*), as well as recent taxonomic changes (see Table 1).

We compiled a list of 17 species (three amphibians, 14 reptiles) potentially occurring in Sonora (Table 4) based on species for which undocumented observations in Sonora exist but for which museum or other records are not available, and on species that have not been recorded or observed in the state, but whose distributional ranges come close to the borders of Sonora. We did not include these species in our analyses and summaries.

### General distribution

Fourteen of the 38 species of amphibians that inhabit Sonora are endemic to Mexico, one of which is restricted to small areas in the Sierra Madre Occidental in eastern Sonora and western Chihuahua (Table 2). Four more are distributed in the Sierra Madre Occidental mainly in the states of Chihuahua, Durango, Sinaloa, and Sonora (Table 2). Another six species are distributed along the Pacific coast, and three more along the Pacific coast extending eastward through the Balsas depression, with one of these three even reaching the state of Veracruz (Table 2). Of the 24 amphibian species not endemic to Mexico that inhabit Sonora, two are introduced species, 17 more are found in the US and Mexico, and the remaining five species have a wide distribution from Canada to Central America, from the US to Central or South America, or from Mexico to Central or South America (Table 2).

The American Crocodile (*Crocodylusacutus*) is widely distributed from the eastern US to South America. One of the seventeen species of turtles that inhabit the state is endemic to Sonora (Table 2). Five more are endemic to Mexico. Of the eleven species of turtles not endemic to Mexico that occur in Sonora, one is introduced. Four more are distributed from the US to Mexico, one more is found from Mexico to Central America, and the remaining five species have a circumtropical or circumglobal distribution (Table 2). Fourteen of the 69 species of lizards that occur in the state are endemic to Sonora, nine of them to islands of the Gulf of California. Thirteen more are endemic to Mexico (Table 2). Of the 42 lizard species not endemic to Mexico that inhabit Sonora, three are introduced, 38 more are found in the US and Mexico, and the remaining species have a wide distribution that includes Mexico and South America (*Phyllodactylustuberculosus*) (Table 2). Two of the 75 species of snakes that inhabit the state are endemic to Sonoran islands of the Gulf of California (Table 2). Another 21 snake species that are found in Sonora are endemic to Mexico. Of the 52 snake species not endemic to Mexico that occur in Sonora, one is introduced, 41 more are distributed from the US to Mexico, six more range from the US or Canada to Central or even South America, and three more are found from Mexico to Central or South America (Table 2).

**Table 2.** Amphibians and reptiles of Sonora with distributional and conservation status. Ecoregion (1 = Western mainland deserts; 2 = High northeastern valleys; 3 = Eastern mountains; 4 = Subtropical lowlands and foothills; 5 = Marine; 6 = Islands); IUCN Status (DD = Data Deficient; LC = Least Concern, VU = Vulnerable, NT = Near Threatened; EN = Endangered; CE = Critically Endangered; NE = not Evaluated) according to the IUCN Red List (The IUCN Red List of Threatened Species, Version 2018-1; www.iucnredlist.org; accessed 14 September 2018), conservation status in Mexico according to SEMARNAT (2010) (P = in danger of extinction, A = threatened; Pr = subject to special protection, NL – not listed), and Environmental Vulnerability Score (EVS – the higher the score the greater the vulnerability: low (L) vulnerability species (EVS of 3–9); medium (M) vulnerability species (EVS of 10–13); and high (H) vulnerability species (EVS of 14–20) from Wilson et al. (2013a,b) and Johnson et al. (2015). Global Distribution (GD): 0 = Endemic to Sonora; 1 = Endemic to Mexico; 2 = Shared between the US and Mexico; 3 = widely distributed from Canada or the US to Central or South America; 4 = widely distributed from Mexico to Central America; 5 = circumglobal distribution; 6 = Pacific and Indian Oceans; IN = Introduced to Sonora. Source of first record (year in parentheses) is the voucher specimen (see Appendix 1 for abbreviations) or paper associated with the first documentation of a species in Sonora.

	IUCN	EVS	SEMARNAT	Ecoregions	GD	Source of first record
**Class Amphibia**						
**Order Anura**						
** Bufonidae **						
*Anaxyruscognatus* (Say, 1823)	LC	L (8)	NL	1, 2	2	UAZ 08894 (1957)
*Anaxyrusdebilis* (Girard, 1854)	LC	L (7)	Pr	2	2	UAZ 40063 (1974)
*Anaxyruskelloggi* (Taylor, 1938)	LC	H (14)	NL	1, 4	1	UTEP H-14419 (1955)
*Anaxyrusmexicanus* (Brocchi, 1879)	NT	M (13)	NL	3	1	UAZ 15045 (1953)
*Anaxyruspunctatus* (Baird & Girard, 1852)	LC	L (5)	NL	1, 2, 3, 4, 6	2	UAZ 16973 (1905)
*Anaxyrusretiformis* (Sanders & Smith, 1951)	LC	M (12)	Pr	1	2	MCZ A-48217 (1700)
*Anaxyruswoodhousii* (Girard, 1854)	LC	M (10)	NL	1, 2	2	USNM 2536 (1855)
*Inciliusalvarius* (Girard, 1859)	LC	M (11)	NL	1, 2, 3, 4	2	USNM 21063 (1893)
*Inciliusmarmoreus* (Wiegmann, 1833)	LC	M (11)	NL	4	1	UAZ 57334-PSV (2011)
*Inciliusmazatlanensis* (Taylor, 1940)	LC	M (12)	NL	1, 2, 3, 4	1	UAZ 11817 (1953)
*Inciliusmccoyi* Santos-Barrera & Flores-Villela, 2011	NE	H (14)	NL	3	1	UAZ 28229 (1964)
*Rhinellahorribilis* (Wiegmann, 1833)	NE	NE	NL	1, 4	3	USNM 47243 (1898)
** Craugastoridae **						
*Craugastoraugusti* (Dugès, 1879)	LC	L (8)	NL	3, 4	2	USNM311989 (1921)
*Craugastoroccidentalis* (Taylor, 1941)	DD	M (13)	NL	3, 4	1	AMNH A-84437 (1970)
*Craugastortarahumaraensis* (Taylor, 1940)	VU	H (17)	Pr	3	1	UAZ 28133 (1968)
** Eleutherodactylidae **						
*Eleutherodactylusinterorbitalis* (Langebartel & Shannon, 1956)	DD	H (15)	Pr	3, 4	1	UAZ 56549-PSV (2005)
** Hylidae **						
*Agalychnisdacnicolor* (Cope, 1864)	LC	M (13)	NL	3, 4	1	LACM 90158 (1960)
*Dryophytesarenicolor* Cope, 1886	LC	L (7)	NL	2, 3, 4	2	MVZ 28776 (1939)
*Dryophyteswrightorum* (Taylor, 1939)	LC	L (9)	NL	2, 3	2	BYU 34818 (1979)
*Smiliscabaudinii* (Duméril & Bibron, 1841)	LC	L (3)	NL	4	3	MVZ 50460 (1950)
*Smiliscafodiens* (Boulenger, 1882)	LC	L (8)	NL	1, 4	2	UMMZ 72186 (1932)
*Tlalocohylasmithii* (Boulenger, 1902)	LC	M (11)	NL	1, 4	1	UAZ 16066 (1956)
** Leptodactylidae **						
*Leptodactylusmelanonotus* (Hallowell, 1861)	LC	L (6)	NL	1, 2, 4	4	MVZ 26066 (1938)
** Microhylidae **						
*Gastrophrynemazatlanensis* (Taylor, 1943)	NE	L (8)	NL	1, 3, 4	2	UMMZ 72177 (1932)
*Hypopachusvariolosus* (Cope, 1866)	LC	L (4)	NL	4	3	UAZ 47259 (1938)
** Ranidae **						
*Ranaberlandieri* Baird, 1859	N/A	N/A	N/A	N/A	IN	ASU HP-00020-21 (2006)
*Ranacatesbeiana* Shaw, 1802	N/A	N/A	N/A	N/A	IN	CAS SUA 202273 (1955)
*Ranachiricahuensis* Platz & Mecham, 1979	VU	M (11)	A	2, 3	2	LACM 91589 (1965)
*Ranaforreri* Boulenger, 1883	LC	L (3)	Pr	1, 4	4	KUH 37904 (1954)
*Ranamagnaocularis* Frost & Bagnara, 1976	LC	M (12)	NL	1, 2, 3, 4	1	CAS SUA 15580 (1955)
*Ranapustulosa* Boulenger, 1883	LC	L (3)	Pr	4	1	ASNHC 13774 (1969)
*Ranatarahumarae* Boulenger, 1917	VU	L (8)	NL	3	2	UMMZ 154302 (1935)
*Ranayavapaiensis* Platz & Frost, 1984	LC	M (12)	Pr	1, 3, 4	2	CAS SUA 10295 (1950)
** Scaphiopodidae **						
*Scaphiopuscouchi* Baird, 1854	LC	L (3)	NL	1, 2, 3, 4	2	Allen, 1933 (1932)
*Speamultiplicata* (Cope, 1863)	LC	L (6)	NL	1, 2, 3	2	USNM 21801 (1894)
**Order Caudata**						
** Ambystomatidae **						
*Ambystomamarvortium* Baird, 1850	LC	M (10)	NL	1, 2	2	UMMZ 78353 (1935)
*Ambystomarosaceum* Taylor, 1941	LC	H (14)	Pr	3	1	USNM 17352 (1891)
** Plethodontidae **						
*Isthmurasierraoccidentalis* (Lowe, Jones, & Wright, 1968)	NE	NE	NL	3	1	LACM 39200 (1964)
**Class Reptilia**						
**Order Crocodylia**						
** Crocodylidae **						
*Crocodylusacutus* Cuvier, 1807	VU	H (14)	Pr	5	3	PBDB 20495 (1764)
**Order Squamata**						
**Suborder Lacertilia**						
** Anguidae **						
*Elgariakingii* Gray, 1838	LC	M (10)	Pr	2, 3	2	UAZ 07265 (1905)
** Crotaphytidae **						
*Crotaphytuscollaris* (Say, 1823)	LC	M (13)	A	2, 3	2	CAS HERP 3411 (1892)
*Crotaphytusdickersonae* Schmidt, 1922	LC	H (16)	NL	1, 6	0	CAS HERP 53264 (1921)
*Crotaphytusnebrius* Axtell & Montanucci, 1977	LC	M (12)	NL	1, 3	2	MVZ 10164 (1926)
*Gambeliawislizenii* (Baird & Girard, 1852)	LC	M (13)	Pr	1, 2	2	USNM 43183 (1910)
** Dactyloidae **						
*Anolisnebulosus* (Wiegmann, 1834)	LC	M (13)	NL	3, 4	1	MVZ 84691 (1818)
** Eublepharidae **						
*Coleonyxfasciatus* (Boulenger, 1885)	LC	H (17)	NL	3, 4	1	UAZ 01186 (1958)
*Coleonyxvariegatus* (Baird, 1858)	LC	M (11)	Pr	1, 2, 4	2	UCM 58228 (1800)
**Gekkonidae (Introduced)**						
*Hemidactylusfrenatus* Schlegel, 1836	N/A	N/A	N/A	N/A	IN	UABC 1728 (2007)
*Hemidactylusturcicus* (Linnaeus, 1758)	N/A	N/A	N/A	N/A	IN	UAZ 56726-PSV (2007)
** Helodermatidae **						
*Helodermaexasperatum* Bogert and Martín del Campo, 1856	NE	NE	NL	3, 4	1	LACM 62549 (1942)
*Helodermasuspectum* Cope, 1869	NT	H (15)	A	1, 2, 3, 4	2	USNM 20998 (1893)
** Iguanidae **						
*Ctenosauraconspicuosa* Dickerson, 1919	NE	H (16)	NL	6	0	CAS HERP 55034 (1912)
*Ctenosauramacrolopha* Smith, 1972	NE	H (19)	NL	1, 3, 4	1	SDNHM 3859 (1930)
*Ctenosauranolascensis* Smith, 1972	VU	H (17)	NL	6	0	CAS HERP 50562 (1921)
*Dipsosaurusdorsalis* (Baird & Girard, 1852)	LC	M (11)	NL	1	2	MVZ 20843 (1936)
*Sauromalusater* Duméril, 1856	LC	M (13)	Pr	1	2	USNM 13483 (1883)
*Sauromalushispidus* Stejneger, 1891	N/A	N/A	N/A	N/A	IN	CAS HERP 104443 (1967)
*Sauromalusvarius* Dickerson, 1919	NE	H (16)	A	6	10	USNM 64441 (1911)
** Phrynosomatidae **						
*Callisaurusdraconoides* Blainville, 1835	LC	M (12)	A	1, 4	2	CAS HERP 55037 (1911)
*Cophosaurustexanus* Troschel, 1852	LC	H (14)	A	1, 2, 3	2	CAS SUR 9882 (1942)
*Holbrookiaapproximans* Baird, 1859	NE	H (14)	NL	1	1	UCM 58250 (1800)
*Holbrookiaelegans* Bocourt, 1874	LC	M (13)	NL	1, 2, 3, 4	2	MCZ R-641 (1859)
*Phrynosomacornutum* (Harlan, 1825)	LC	M (11)	NL	2	2	MVZ 38192 (1818)
*Phrynosomaditmarsi* Stejneger, 1906	DD	H (16)	NL	3	0	USNM 36013 (1897)
*Phrynosomagoodei* Stejneger, 1893	NE	M (13)	NL	1	2	CM S4812 (1928)
*Phrynosomahernandesi* Girard, 1858	LC	M (13)	NL	2, 3	2	USNM 21022 (1893)
*Phrynosomamcallii* (Hallowell, 1852)	NT	H (15)	A	1	2	USNM 21841 (1894)
*Phrynosomamodestum* Girard, 1852	LC	M (12)	NL	2	2	USNM 21021 (1893)
*Phrynosomaorbiculare* (Linnaeus, 1766)	LC	M (12)	A	3	1	MCZ R-169820 (1700)
*Phrynosomasolare* Gray, 1845	LC	H (14)	NL	1, 2, 3, 4, 6	2	UAZ 02189 (1905)
*Sceloporusalbiventris* Smith, 1939	NE	H (16)	NL	3, 4	1	BYU 21179 (1961)
*Sceloporusclarkii* Baird & Girard, 1852	LC	M (10)	NL	1, 2, 3, 4, 6	2	CAS HERP 50516 (1921)
*Sceloporuscowlesi* Lowe & Norris, 1956	NE	M (13)	NL	2	2	UAZ 36545 (1973)
*Sceloporusjarrovii* Cope, 1875	NE	M (11)	NL	3	2	USNM 17252 (1891)
*Sceloporuslemosespinali* Lara-Góngora, 2004	DD	H (16)	NL	3	1	UAZ 16588 (1966)
*Sceloporusmagister* Hallowell, 1854	LC	L (9)	NL	1	2	CAS HERP 53359 (1921)
*Sceloporusnelsoni* Cochran, 1923	LC	M (13)	NL	3, 4	1	MVZ 28914 (1939)
*Sceloporuspoinsettii* Baird & Girard, 1852	LC	M (12)	NL	3	2	USNM 313440 (1921)
*Sceloporusslevini* Smith, 1937	LC	M (11)	NL	2, 3	2	UAZ 02914 (1953)
*Sceloporusvirgatus* Smith, 1938	LC	H (15)	NL	3	2	MCZ R-46525 (1933)
*Umarufopunctata* Cope, 1895	NT	H (16)	NL	1	2	CAS HERP 53368 (1921)
*Urosaurusbicarinatus* (Duméril, 1856)	LC	M (12)	NL	4	1	MVZ 28889 (1939)
*Urosaurusgraciosus* Hallowell, 1854	LC	H (14)	NL	1	2	MVZ 10160 (1926)
*Urosaurusornatus* (Baird & Girard, 1852)	LC	M (10)	NL	1, 2, 3, 4, 6	2	CAS HERP 53257 (1921)
*Utanolascensis* Van Denburgh & Slevin, 1921	LC	H (17)	A	6	0	CAS HERP 50539 (1921)
*Utapalmeri* Stejneger, 1890	VU	H (17)	A	6	0	CAS HERP 50580 (1921)
*Utastansburiana* Baird & Girard, 1852	LC	M (11)	A	1, 6	2	CAS HERP 50705 (1921)
** Phyllodactylidae **						
*Phyllodactylushomolepidurus* Smith, 1935	LC	H (15)	Pr	1, 4	1	CMNH 13022 (1932)
*Phyllodactylusnocticolus* Dixon, 1964	NE	M (10)	NL	6	2	CAS HERP 50798 (1921)
*Phyllodactylusnolascoensis* Dixon, 1964	NE	NE	NL	6	0	CAS HERP 50550 (1921)
*Phyllodactylustuberculosus* Wiegmann, 1835	LC	L (8)	NL	4	4	KUH 24117 (1948)
** Scincidae **						
*Plestiodoncallicephalus* (Bocourt, 1879)	LC	M (12)	NL	3	2	UAZ 03469 (1905)
*Plestiodonobsoletus* (Baird & Girard, 1852)	LC	M (11)	NL	1, 3	2	UAZ 35168 (1972)
*Plestiodonparviauriculatus* (Taylor, 1933)	DD	H (15)	Pr	3, 4	1	USNM 47536 (1899)
** Teiidae **						
*Aspidoscelisbacatus* (Van Denburgh & Slevin, 1921)	LC	H (17)	Pr	6	0	Van Denburgh and Slevin 1921 (1921)
*Aspidoscelisburti* (Taylor, 1938)	LC	H (15)	NL	1	0	CAS HERP 53425 (1921)
*Aspidosceliscostatus* (Cope, 1878)	NE	M (11)	Pr	1, 3, 4	1	MVZ 28921 (1939)
*Aspidoscelisestebanensis* (Dickerson, 1919)	NE	NE	Pr	6	0	Dickerson, 1919 (1919)
*Aspidoscelisexsanguis* (Lowe, 1956)	LC	H (14)	NL	3	2	MVZ 21018 (1936)
*Aspidoscelismartyris* (Stejneger, 1891)	VU	H (17)	Pr	6	0	Stejneger, 1891 (1891)
*Aspidoscelisopatae* (Wright, 1967)	DD	H (16)	NL	3	0	UAZ 09228 (1963)
*Aspidoscelissonorae* (Lowe & Wright, 1964)	LC	M (13)	NL	1, 2, 3	2	UAZ 05045 (1905)
*Aspidoscelisstictogrammus* (Burger, 1950)	NE	H (14)	NL	1, 3	2	USNM 15752 (1889)
*Aspidoscelistigris* (Baird & Girard, 1852)	LC	L (8)	NL	1	2	CAS HERP 49152 (1921)
*Aspidoscelisuniparens* (Wright & Lowe, 1965)	LC	H (15)	NL	2	2	UAZ 05125 (1905)
*Aspidoscelisxanthonotus* (Duellman & Lowe, 1953)	NE	H (14)	NL	1	2	Rosen and Quijada-Mascareñas 2009 (2009)
** Xantusidae **						
*Xantusiajaycolei* Bezy, Bezy, & Bolles, 2009	NE	H (16)	NL	1	0	UAZ 10760 (1964)
*Xantusiavigilis* Baird, 1859	LC	NE	NL	1	2	CAS HERP 84144 (1949)
**Suborder Serpentes**						
** Boidae **						
*Boasigma* Smith, 1943	NE	NE	NL	1, 3, 4	1	USNM 61956 (1887)
*Lichanuratrivirgata* Cope, 1861	LC	M (10)	A	1	2	SDNHM 10793 (1933)
** Colubridae **						
*Arizonaelegans* Kennicott, 1859	LC	L (5)	NL	1	2	SDNHM 16479 (1934)
*Chilomeniscusstramineus* Cope, 1860	LC	L (8)	Pr	1, 6	2	UAZ 23194 (1958)
*Chionactisannulata* (Baird, 1858)	LC	M (12)	NL	1	2	CUMV 1243 (1930)
*Chionactispalarostris* (Klauber, 1937)	LC	M (13)	NL	1	2	MCZ R-36890 (1932)
*Drymarchonmelanurus* (Duméril,Bibron & Duméril, 1854)	LC	L (6)	NL	1, 3, 4	3	
*Drymobiusmargaritiferus* (Schlegel, 1837)	NE	L (6)	NL	4	3	MVZ 28930 (1939)
*Gyalopioncanum* Cope, 1861	LC	L (9)	NL	2, 3	2	UAZ 20736 (1954)
*Gyalopionquadrangulare* (Günther, 1893)	LC	M (11)	Pr	1, 4	2	KUH 24113 (1948)
*Lampropeltiscaliforniae* (Blainville, 1835)	NE	M (10)	NL	1	2	UAZ 25105 (1905)
*Lampropeltisknoblochi* Taylor, 1940	NE	M (10)	NL	3	2	SDNHM 41106 (1950)
*Lampropeltisnigrita* Zweifel & Norris, 1955	NE	NE	NL	1, 2, 3, 4, 6	2	USNM 21720 (1894)
*Lampropeltispolyzona* Cope, 1860	NE	L (7)	NL	3, 4	1	MVZ 50813 (1950)
*Lampropeltissplendida* (Baird & Girard, 1853)	NE	M (12)	NL	2, 3	2	Baird and Girard 1853 (1853)
*Leptophisdiplotropis* (Günther, 1872)	LC	H (14)	A	3, 4	1	SDNHM 18176 (1947)
*Masticophisbilineatus* Jan, 1863	LC	M (11)	NL	1, 2, 3, 4, 6	2	USNM 15880 (1889)
*Masticophisflagellum* Shaw, 1802	LC	L (8)	A	1, 2, 3, 4, 6	2	USNM 56759 (1902)
*Masticophismentovarius* (Duméril, Bibron & Duméril, 1854)	LC	L (6)	A	3,4	4	SDNHM 18183 (1947)
*Masticophisslevini* Lowe & Norris, 1955	LC	H (17)	NL	6	0	SDNHM 3826 (1930)
*Mastigodryascliftoni* (Hardy, 1964)	NE	H (14)	NL	4	1	UAZ 42231 (1975)
*Oxybelisaeneus* (Wagler, 1824)	NE	L (5)	NL	1, 3, 4	3	SDNHM 18189 (1947)
*Phyllorhynchusbrowni* Stejneger, 1890	LC	M (13)	Pr	1, 4	2	MVZ 50740 (1950)
*Phyllorhynchusdecurtatus* (Cope, 1868)	LC	M (11)	NL	1	2	MVZ 10170 (1926)
*Pituophiscatenifer* (Blainville, 1835)	LC	L (9)	NL	1, 2, 3, 4, 6	2	MVZ 5886 (1915)
*Pituophisdeppei* (Duméril, 1853)	LC	H (14)	A	3	1	T.R. Van Devender (son-trv-5147) (1997)
*Pseudoficimiafrontalis* (Cope, 1864)	LC	M (13)	NL	4	1	UAZ 21338 (1967)
*Rhinocheiluslecontei* Baird & Girard, 1853	LC	L (8)	NL	1, 2, 3, 4	2	UMMZ 75636 (1933)
*Salvadorabairdii* Jan & Sordelli, 1860	LC	H (15)	Pr	3	1	AMNH 102194 (1968)
*Salvadoradeserticola* Schmidt, 1940	NE	H (14)	NL	1, 2, 3	2	MVZ 21029 (1936)
*Salvadoragrahamiae* Baird & Girard, 1853	LC	M (10)	NL	2, 3	2	UAZ 26182 (1952)
*Salvadorahexalepis* (Cope, 1867)	LC	M (10)	NL	1	2	UAZ 26300 (1905)
*Senticolistriaspis* (Cope, 1866)	LC	L (6)	NL	1, 3, 4	3	CAS HERP 63101 (1928)
*Sonoraaemula* (Cope, 1879)	NT	H (16)	Pr	3, 4	1	MPM H 6448 (1900)
*Sonorasemiannulata* Baird & Girard, 1853	LC	L (5)	NL	1, 2	2	UAZ 26340 (1953)
*Sympholislippiens* Cope, 1862	NE	H (14)	NL	4	1	MVZ 76333 (1963)
*Tantillahobartsmithi* Taylor, 1936	LC	M (11)	NL	1, 2, 3	2	LACM 20473 (1950)
*Tantillawilcoxi* Stejneger, 1902	LC	M (10)	NL	3	2	UAZ 28201 (1964)
*Tantillayaquia* Smith, 1942	LC	M (10)	NL	1, 3, 4	2	SDNHM 18190 (1947)
*Trimorphodonlambda* Cope, 1886	NE	M (13)	NL	1, 3, 4, 6	2	USNM 56321 91902)
*Trimorphodontau* Cope, 1870	LC	M (13)	NL	3, 4	1	UAZ 27070 (1905)
** Dipsadidae **						
*Coniophaneslateritius* Cope, 1862	DD	M (13)	NL	4	1	Ambía Molina 1969 (1969)
*Diadophispunctatus* (Linnaeus, 1766)	LC	L (4)	NL	1, 2, 3	2	UAZ 24162 (1905)
*Geophisdugesii* Bocourt, 1883	LC	M (13)	NL	3	1	Enderson and Bezy 2007 (2007)
*Heterodonkennerlyi* Kennicott, 1860	NE	M (11)	Pr	2	2	USNM 1253 (1855)
*Hypsiglenachlorophaea* Cope, 1860	NE	L (8)	NL	1, 2, 3, 4, 6	2	Allen 1933 (1932)
*Imantodesgemmistratus* (Cope, 1861)	NE	L (6)	Pr	4	4	UAZ 50923 (1905)
*Leptodeirapunctata* (Peters, 1866)	LC	H (17)	NL	4	1	CAS HERP 93855 (1962)
*Leptodeirasplendida* Günther, 1895	LC	H (14)	NL	3, 4	1	MVZ 50835 (1950)
*Tropidodipsasrepleta* Smith, Lemos-Espinal, Hartman & Chiszar, 2005	DD	H (17)	NL	3, 4	1	UCM 65700 (2003)
** Elapidae **						
*Hydrophisplaturus* (Linnaeus, 1766)	LC	NE	NL	5	6	UAZ 39726 (1962)
*Micruroideseuryxanthus* (Kennicott, 1860)	LC	H (15)	A	1, 3, 4, 6	2	UMMZ 78434 (1935)
*Micrurusdistans* (Kennicott, 1860)	LC	H (14)	Pr	3, 4	1	MVZ 28933 (1939)
** Leptotyphlopidae **						
*Renahumilis* Baird & Girard, 1853	LC	L (8)	NL	1, 3, 4	2	USNM 141978 (1957)
** Natricidae **						
*Storeriastorerioides* (Cope, 1865)	LC	M (11)	NL	3	1	UAZ 28125 (1964)
*Thamnophiscyrtopsis* (Kennicott, 1860)	LC	L (7)	A	1, 2, 3, 4	3	USNM 21056 (1893)
*Thamnophiseques* (Reuss, 1834)	LC	L (8)	A	1, 2, 3, 4	2	MCZ R-5891 (1700)
*Thamnophismarcianus* (Baird & Girard, 1853)	LC	M (10)	A	1, 2, 3	3	USNM 21822 (1894)
*Thamnophismelanogaster* (Peters, 1864)	EN	H (15)	A	3	1	BYU 13505 (1956)
*Thamnophisunilabialis* Tanner, 1985	NE	NE	NL	3	1	USNM 21055 (1893)
*Thamnophisvalidus* (Kennicott, 1860)	NE	M (12)	NL	4	1	KUH 47567 (1959)
** Typhlopidae **						
*Indotyphlopsbraminus* (Daudin, 1803)	N/A	N/A	N/A	N/A	IN	MZFC 6147 (1991)
** Viperidae **						
*Agkistrodonbilineatus* (Günther, 1863)	NT	M (11)	Pr	4	4	SDNHM 40270 (1949)
*Crotalusatrox* Baird & Girard, 1853	LC	L (9)	Pr	1, 2, 3, 6	2	USNM 21045 (1893)
*Crotalusbasiliscus* (Cope, 1864)	LC	H (16)	Pr	1, 4	1	SDNHM 18181 (1947)
*Crotaluscerastes* Hallowell, 1854	LC	H (16)	Pr	1	2	CAS HERP 81515 (1947)
*Crotalusestebanensis* (Klauber, 1949)	LC	H (19)	NL	6	0	USNM 64586 (1911)
*Crotaluslepidus* (Kennicott, 1861)	LC	M (12)	Pr	3	2	SDNHM 42906 (1952)
*Crotalusmolossus* Baird & Girard, 1853	LC	L (8)	Pr	1, 2, 3, 4, 6	2	SDNHM 3445 (1932)
*Crotaluspricei* Van Denburgh, 1895	LC	H (14)	Pr	3	2	UMMZ 78456 (1935)
*Crotaluspyrrhus* (Cope, 1866)	NE	NE	NL	1	2	UAZ 27600 (1964)
*Crotalusscutulatus* (Kennicott, 1861)	LC	L (11)	Pr	1, 2	2	UAZ 27355 (1930)
*Crotalustigris* Kennicott, 1859	LC	H (16)	Pr	1, 3, 4, 6	2	SDNHM 3237 (1930)
*Crotalusviridis* (Rafinesque, 1818)	LC	M (12)	Pr	2	2	USNM 61955 (1887)
*Crotaluswillardi* Meek, 1905	LC	M (13)	Pr	3	2	UMMZ 78449 (1935)
**Order Testudines**						
** Chelonidae **						
*Carettacaretta* (Linnaeus, 1758)	VU	NE	P	5	5	UAZ 36495 (1954)
*Cheloniamydas* (Linnaeus, 1758)	EN	NE	P	5	5	USNM 21818 (1894)
*Eretmochelysimbricata* (Linnaeus, 1766)	NE	NE	P	5	5	Grismer, 2002 (2002)
*Lepidochelysolivacea* (Eschscholtz, 1829)	VU	NE	P	5	5	SDNHM 49849 (1961)
** Dermochelyidae **						
*Dermochelyscoriacea* (Vandelli, 1761)	VU	NE	P	5	5	UAZ 40133 (1974)
** Emydidae **						
*Terrapenenelsoni* Stejneger, 1925	DD	H (18)	Pr	3, 4	1	SDNHM 42411 (1930)
*Terrapeneornata* (Agassiz, 1857)	NT	H (15)	Pr	2, 3	2	USNM 20993 (1893)
*Trachemysnebulosa* (Van Denburgh, 1895)	NE	H (18)	NL	4	1	UMNH 3823 (1961)
*Trachemysyaquia* (Legler & Webb, 1970)	VU	H (19)	NL	1, 3, 4	0	UMNH 12449 (1963)
** Geoemydidae **						
*Rhinoclemmyspulcherrima* (Gray, 1855)	NE	L (8)	NL	4	4	MVZ 50913 (1950)
** Kinosternidae **						
*Kinosternonalamosae* Berry & Legler, 1980	DD	H (14)	Pr	1, 4	1	MVZ 50907 (1950)
*Kinosternonarizonense* Gilmore, 1922	LC	H (15)	NL	1	2	UMMZ 72234 (1950)
*Kinosternonintegrum* LeConte, 1854	LC	M (11)	Pr	1, 3, 4	1	UMMZ 79514 (1935)
*Kinosternonsonoriense* Le Conte, 1854	NT	H (14)	P	1, 2, 3	2	USNM 20984 (1893)
**Family Testudinidae**						
*Gopherusevgoodei* Edwards, Karl, Vaughn, Rosen, Meléndez-Torres, & Murphy, 2016	NE	NE	NL	3, 4	1	ROM 53301 (1942)
*Gopherusmorafkai* Murphy, Berry, Edwards, Leviton, Lathrop, & Riedle, 2011	NE	H (15)	NL	1, 3, 6	2	USNM 21159 (1894)
** Trionychidae **						
*Apalonespinifera* (Le Sueur, 1827)	N/A	N/A	N/A	N/A	IN	UAZ 56727-PSV (2007)

### Ecoregions

The most diverse Sonora ecoregions in terms of the herpetofauna are the Eastern mountains (54% of the total number of amphibian and reptile species for the state) represented by the Sierra Madre Occidental and associated mountains, and the Western Mainland Desert (49%) represented mainly by the Sonoran Desert (Fig. 4). The Island (16%) and Marine (4%) are the least occupied ecoregions (Table 5). In general, the highest richness of amphibian species is observed in the Subtropical Lowlands and Foothills of the Sierra Madre Occidental with 61% of the total number of species, followed by the Eastern Mountains (58%), the Western Mainland Deserts (50%), and the High Northeastern Valleys (39%). Amphibians are almost absent in the Island ecoregion with only two species recorded (6%) and due to their limitations to inhabit saline environments they are absent in the Marine ecoregion (Table 5). The Subtropical Lowlands and Foothills of the Sierra Madre Occidental had 67% of the anuran species in Sonora, whereas caudate amphibians are absent in this ecoregion showing their highest percentage of presence in the Eastern Mountains with two (67%) of the three species occurring in this ecoregion. In reptiles, the highest species richness is found in the Eastern Mountains (53%) ecoregion. This is the ecoregion with the highest number of snake (61%) and turtle (44%) species, although the same number of turtle species is found in the Subtropical Lowlands and Foothills of the Sierra Madre Occidental. Snakes are also diverse in the Western Mainland Deserts and the Subtropical Lowlands and Foothills of the Sierra Madre Occidental; each of these ecoregions hosts 38 snake species (51% of the total number of snake species recorded in Sonora). On the other hand, due to their conspicuousness and adaptations for arid environments, lizards have their highest diversity in the Western Mainland Deserts (48%) followed by the Eastern Mountains (47%), and they are the most diverse taxonomic group in the Island ecoregion, which is represented by dry environments, with 15 species (23%). Snakes are also diverse in the Island ecoregion with 13 species (18%). This is explained in part by the high vagility, adaptations to dry environments, and speciation rates of these two squamate suborders. Testudines is the taxonomic group with the highest percentage of species (5 = 31% of the total number of turtles in Sonora) in the Marine ecoregion, followed by snakes and crocodilians, both groups with one species representing 1 and 100% of the total number of species in their groups respectively. Five of the species that occur in the Marine ecoregion have a circumglobal or circumtropical distribution (five turtles). The other two species occurring in the Marine ecoregion are a crocodile that was thought until recently to be extirpated from Sonora but may be staging a comeback on the southern coast (Rorabaugh 2017), and a sea snake distributed across the Pacific and Indo-Pacific Oceans. The general reptile pattern of diversity is driven by lizards and snakes, except in the Marine ecoregions which is dominated by sea turtles of the families Cheloniidae and Dermochelyidae (Table 5).

### Comparisons with neighboring states

Overall, Sonora shares the most species with Chihuahua, Sinaloa, and Arizona (Table 6). For amphibians, Sonora shares the most species with Chihuahua and Sinaloa. For reptiles, Sonora shares about half its species with Chihuahua, Sinaloa, and Arizona (Table 6). Previous comparisons of shared herpetofaunal species among neighboring states in the US-Mexico border region found high levels of similarity between Sonora and Chihuahua (Enderson et al. 2009, Smith and Lemos-Espinal 2015, Lemos-Espinal et al. 2017). However, an analysis based on “biogeographic affinity” resulted in Sonora being closest or most similar to Sinaloa (Enderson et al. 2009, Lavín-Murcio and Lazcano 2010). There is some variation, though, in these affinities depending on which specific herpetofaunal taxa are being examined (Enderson et al. 2009). Such a pattern probably reflects the fact that Sonora, Chihuahua, Arizona, and Sinaloa all have extensive tracts of arid habitats. Shared habitats and vegetation types likely lead to similarities in species among Sonora and neighboring states (see also Smith and Lemos-Espinal 2015, Lemos-Espinal and Smith 2016, Lemos-Espinal et al. 2017). The similarity in herpetofauna among three Mexican states and Arizona highlights the necessity for interstate and international approaches to conserving and managing habitats and species (e.g., Grigione et al. 2009, Wiederholt et al. 2013).

### Conservation status

A total of 21 (= 10.9%) species of amphibians and reptiles is IUCN listed (i.e., Vulnerable, Near Threatened, Endangered, or Critically Endangered), but 69 species (= 35.0%) are placed in a protected category by SEMARNAT and 63 species (= 32.6%) are categorized as high risk by the EVS (Tables 3, 5). For amphibians, 11.1% are IUCN listed, 25.0% are protected by SEMARNAT, and 13.8% are at high risk according to the EVS (Tables 3, 5). For reptiles, 10.8% are listed by the IUCN, 38.2% are protected by SEMARNAT, and 36.3% are at high risk according to the EVS (Tables 3, 5). These results suggest that the herpetofauna, especially the reptiles, of Sonora is considered to be of relatively low conservation concern at a global scale, but there is much greater conservation concern at a national level. Indeed, more local assessments (SEMARNAT and EVS) are based on information specific to Mexico and thus are more likely to reflect the conservation needs of the Sonoran herpetofauna (see Lemos-Espinal et al. 2018a,b for a similar assessment for other Mexican states). There are several taxa that, based on their IUCN listing, SEMARNAT category or their EVS, are of conservation concern. Families that include species of particular conservation concern include Bufonidae, Craugastoridae, Eleutherodactylidae, Ranidae, Ambystomidae, Crocodylidae, Helodermatidae, Iguanidae, Phrynosomatidae, Phyllodactylidae, Teiidae, Xantusidae, Colubridae, Dipsadidae, Elapidae, Natricidae, Viperidae, Cheloniidae, Dermochelyidae, Emydidae, Kinosternidae, and Testudinidae (Tables 3, 5). Because the IUCN, SEMARNAT, and EVS categories are based on global or country-level assessments, there are likely amphibians and reptiles whose conservation status in Sonora is not accurately assessed by these measures. Additional assessments at the state level in Sonora, and other Mexican states, are needed to establish conservation or management needs for particular states, or even regions. As an example, frogs in the family Ranidae in Sonora, some of which are considered of conservation concern, are at risk from habitat loss, disease (chytridiomycosis), and predation by introduced species (Rorabaugh and Lemos-Espinal 2016).

**Table 3.** Summary of native species present in Sonora by family, order or suborder, and class. Status summary indicates the number of species found in each IUCN conservation status in the order DD, LC, VU, NT, EN, CE (see Table 2 for abbreviations; in some cases species have not been assigned a status by the IUCN and therefore these may not add up to the total number of species in a taxon) and conservation status in Mexico according to SEMARNAT (2010) in the order NL, Pr, A, and P (see Table 1 for abbreviations). Mean EVS is the mean Environmental Vulnerability Score, scores ≥ 14 are considered high vulnerability (Wilson et al. 2013a, b).

Scientific Name	Genera	Species	IUCN	EVS	SEMARNAT
**Class Amphibia**					
**Order Anura**	**15**	**33**	**2,24,3,1,0,0**	**9.3**	**25,7,1,0**
Bufonidae	3	12	0,9,0,1,0,0	10.6	10,2,0,0
Craugastoridae	1	3	1,1,1,0,0,0	12.7	2,1,0,0
Eleutherodactylidae	1	1	1,0,0,0,0,0	15	0,1,0,0
Hylidae	4	6	0,6,0,0,0,0	8.5	6,0,0,0
Leptodactylidae	1	1	0,1,0,0,0,0	6	1,0,0,0
Microhylidae	2	2	0,1,0,0,0,0	6	2,0,0,0
Ranidae	1	6	0,4,2,0,0,0	8.2	2,3,1,0
Scaphiopodidae	2	2	0,2,0,0,0,0	4.5	2,0,0,0
**Order Caudata**	**2**	**3**	**0,2,0,0,0,0**	**12**	**2,1,0,0**
Ambystomatidae	1	2	0,2,0,0,0,0	12	1,1,0,0
Plethodontidae	1	1	0,0,0,0,0,0		1,0,0,0
**Subtotal**	**17**	**36**	**2,26,3,1,0,0**	**9.4**	**27,8,1,0**
**Class Reptilia**					
**Order Crocodylia**	**1**	**1**	**0,0,1,0,0,0**	**14**	**0,1,0,0**
** Crocodylidae **	1	1	0,0,1,0,0,0	14	0,1,0,0
**Order Squamata**	**60**	**140**	**6,90,3,5,1,0**	**12.2**	**91,29,20,0**
**Suborder Lacertilia**	**21**	**66**	**4,40,3,3,0,0**	**13.5**	**46,10,10,0**
Anguidae	1	1	0,1,0,0,0,0	10	0,1,0,0
Crotaphytidae	2	4	0,4,0,0,0,0	13.5	2,1,1,0
Dactyloidae	1	1	0,1,0,0,0,0	13	1,0,0,0
Eublepharidae	1	2	0,2,0,0,0,0	14	1,1,0,0
Helodermatidae	1	2	0,0,0,1,0,0	15	1,0,1,0
Iguanidae	3	6	0,2,1,0,0,0	15.3	4,1,1,0
Phrynosomatidae	8	29	2,19,1,2,0,0	13.2	22,0,7,0
Phyllodactylidae	1	4	0,2,0,0,0,0	11	3,1,0,0
Scincidae	1	3	1,2,0,0,0,0	12.7	2,1,0,0
Teiidae	1	12	1,6,1,0,0,0	14	8,4,0,0
Xantusidae	1	2	0,1,0,0,0,0	16	2,0,0,0
**Suborder Serpentes**	**39**	**74**	**2,51,0,2,1,0**	**11.1**	**45,19,10,0**
Boidae	2	2	0,1,0,0,0,0	10	1,0,1,0
Colubridae	21	39	0,27,0,1,0,0	10.5	30,5,4,0
Dipsadidae	8	9	2,4,0,0,0,0	11.4	7,2,0,0
Elapidae	3	3	0,3,0,0,0,0	14.5	1,1,1,0
Leptotyphlopidae	1	1	0,1,0,0,0,0	8	1,0,0,0
Natricidae	2	7	0,4,0,0,1,0	10.5	3,0,4,0
Viperidae	2	13	0,11,0,1,0,0	13.1	2,11,0,0
**Order Testudines**	**10**	**16**	**2,2,4,2,1,0**	**14.7**	**6,4,0,6**
Cheloniidae	4	4	0,0,2,0,1,0		0,0,0,4
Dermochelyidae	1	1	0,0,1,0,0,0		0,0,0,1
Emydidae	2	4	1,0,1,1,0,0	17.5	2,2,0,0
Geoemydidae	1	1	0,0,0,0,0,0	8	1,0,0,0
Kinosternidae	1	4	1,2,0,1,0,0	13.5	1,2,0,1
Testudinidae	1	2	0,0,0,0,0,0	15	2,0,0,0
**Subtotal**	**71**	**157**	**8,93,8,7,2,0**	**12.4**	**97,34,20,6**
**Total**	**88**	**193**	**10,119,11,8,2,0**	**11.9**	**124,42,21,6**

To help determine which ecoregions within Sonora support species of particular conservation concern, we summarized the conservation status of reptile and amphibian taxa in each ecoregion found in Sonora (Tables 2, 3). In regard to IUCN categories, none of the amphibians in the Western Mainland Deserts, Subtropical Lowlands and Foothills of the Sierra Madre Occidental, and Island ecoregions are listed; however, one species (2.8%) in the High Northeastern Valleys, and three (8.3%) in the Eastern Mountains ecoregions are included. For SEMARNAT categories, 16.7% of amphibians in the Western Mainland Deserts ecoregion, 14.3% in the High Northeastern Valleys ecoregion, 28.6% in the Eastern Mountains ecoregion, and 18.2% in the Subtropical Lowlands and Foothills of the Sierra Madre Occidental ecoregion are listed. For EVS, 44.4% of the amphibians in the Western Mainland Deserts ecoregion were in the low and medium categories, and 5.6%, represented by only one species, was in the high category; the remaining 5.6% are represented by a species not evaluated. More than half (57.1%) of the amphibians in the High Northeastern Valleys ecoregion are in the low category, and 42.9% are in the medium category; no species in this ecoregion is in the high category. In the Eastern Mountains ecoregion, 38.1% of amphibian species are in the low and medium categories, 19.0% in the high, and the remaining 4.8% are represented by a species not evaluated. For the Subtropical Lowlands and Foothills of the Sierra Madre Occidental ecoregion, 50.0% are in the low category, 36.4% are in the medium category, and 9.1% are in the high category; the remaining 4.5% are represented by a species not evaluated. For the Island ecoregion, the two species occurring in this ecoregion are in the low category.

**Table 4.** List of amphibians and reptiles that could potentially occur in Sonora.

**Class Amphibia**	
**Order Anura**	
** Craugastoridae **	
*Craugastorvocalis* (Taylor, 1940)	Likely to occur in tropical deciduous forest and montane woodlands in the Río Fuerte drainage of extreme southeastern Sonora.
** Ranidae **	
*Ranablairi* (Mecham, Littlejohn,Oldham, Brown, & Brown, 1973)	Likely to occur in Chihuahuan Desert or semi- desert grassland of northeastern Sonora, along the US-Mexico border east of Naco.
** Scaphiopodidae **	
*Speabombifrons* (Cope, 1863)	Likely to occur in Chihuahua desertscrub east and plains grassland of northeastern Sonora.
**Class Reptilia**	
**Order Squamata**	
**Suborder Amphisbaenia**	
*Bipesbiporus* (Cope, 1894)	This species has been observed in the San Carlos Bay, municipality of Guaymas (Ballinger pers. comm., May 2009), but no museum record or voucher exist to support its presence in Sonora.
**Suborder Lacertilia**	
** Anguidae **	
*Barisialevicolis* (Smith, 1942)	Likely to occur in woodlands of the Sierra Madre Occidental of eastern and northeastern Sonora
** Phrynosomatidae **	
*Sceloporusbimaculosus* Phelan & Brattstrom, 1955	Expected in Chihuahuan desertscrub and semi- desert grassland valleys as well as the lower slopes of the mountains along the US – Mexico border from the Río San Pedro valley east to the Sierra San Luis, and potentially in Plains grassland in the southern Animas Valley (northeastern Sonora).
** Scincidae **	
*Plestiodonmultilineatus* (Tanner, 1957)	Likely to occur in woodland of the Sierra Madre Occidental of eastern and northeastern Sonora
**Suborder Serpentes**	
** Boidae **	
*Lichanuraorcutti* Stejneger, 1889	Has been found within a few km of the Sonora border in the Tinajas Altas Mountains of Yuma County, Arizona
** Colubridae **	
*Lampropeltisgentilis* (Baird & Girard, 1853)	Occurs in southeastern Cochise County, Arizona
*Tantillanigriceps* Kennicott, 1860	Likely occurs in northeastern Sonora in Chihuahuan desertscrub or semi-desert grassland from Agua Prieta east to the Sierra San Luis and possibly in Plains grassland in the southern Animas Valley.
** Dipsadidae **	
*Hypsiglenajani* Duges, 1865	Likely to occur in tropical deciduous forest and scrubland of southeastern Sonora.
*Hypsiglenatorquata* (Günther, 1860)	Likely to occur in tropical deciduous forest and scrubland of southeastern Sonora. Mulcahy et al. (2014) suggested the snakes in this area might be an undescribed species of *Hypsiglena*.
*Rhadinaealaureata* (Günther, 1868)	Likely to occur in woodlands of the Sierra Madre Occidental of eastern and northeastern Sonora
** Leptotyphlopidae **	
*Renadissecta* (Cope, 1896)	Expected in Chihuahuan desertscrub, semi-desert grasslands, and into the lower slopes of adjacent mountains along the United States - Mexico border from the Río San Pedro Valley east to the Sierra San Luis, and also in Plains grassland in the southern Animas Valley.
** Natricidae **	
*Thamnophiselegans* (Baird & Girard, 1853)	This species might occur in the Sierras Huachinera and Bacadehuachi and possibly elsewhere in the eastern mountains of Sonora near the Chihuahua border.
** Viperidae **	
*Sistrurustergeminus* (Say, 1823)	Could potentially be found in grasslands along the US – Mexico border from the Río San Pedro Valley east to the Sierra San Luis.
**Order Testudines**	
** Emydidae **	
*Trachemysscripta* (Thunberg, 1792)	This aquatic turtle occurs sparingly as an introduced species in the Colorado River near Yuma, Arizona and in the San Pedro River Valley of Arizona. It could be present along wetted reaches of the Río Colorado in Sonora or in agricultural canals and ditches in that region, and in the Río San Pedro of Sonora near the border with Arizona.

For the IUCN listings, all ecoregions, except the Marine ecoregion, have relatively few species of reptiles in the protected categories (Western Mainland Deserts [5 = 6.6%], High Northeastern Valleys [3 = 7.5%], Eastern Mountains [6 = 7.2%], Subtropical Lowlands and Foothills of the Sierra Madre Occidental [4 = 6.6%], and Island [3 = 10.5%]). Nearly all of the reptiles in the Marine ecoregion (6 = 85.7%) are in the protected categories. However, for the IUCN listing a total of 38 reptile species have not been evaluated, most of them are species recently described or not recognized by the IUCN as populations that deserve species status, but all of them are species with a narrow distribution, which increases their vulnerability. On the other hand, 36.8% of reptiles in the Western Mainland Deserts region, 42.5% from the High Northeastern Valleys ecoregion, 35.4% from the Eastern Mountains ecoregion, 37.1% from the Subtropical Lowlands and Foothills of the Sierra Madre Occidental ecoregion, 85.7% of the Marine ecoregion, and 41.4% from the Island ecoregion are in the protected SEMARNAT categories. For the Western Mainland Deserts ecoregion, 26.3% of the reptiles are in the low EVS category, 36.8% in the medium, and 32.9% in the high; the remaining 3.9% are represented by three species not evaluated. In the High Northeastern Valleys ecoregion, 27.5% of the reptiles are in the low, 47.5% in the medium, and 22.5% in the high category; the remaining 2.5% are represented by a species not evaluated. Of the reptiles in the Eastern Mountains ecoregion, 19.5% are in the low, 39.0% in the medium, and 35.4% in the high category; the remaining 6.1% are represented by five species not evaluated. For the Subtropical Lowlands and Foothills of the Sierra Madre Occidental, 27.4% are in the low EVS category, 32.3% in the medium, and 33.9% in the high; the remaining 6.5% are represented by four species not evaluated. Of the seven reptile species that occur in the Marine ecoregion, only one (14.3%) is in the high category; the other six species (85.7%) are species that have not been evaluated. In the Island ecoregion, 17.2% are in the low EVS category, 24.1% in the medium, and 48.3% in the high; the remaining 10.3% are represented by three species not evaluated. Thus, the reptiles in the Marine ecoregion are clearly the most threatened of the Sonoran herpetofauna.

**Table 5.** Summary of the number of native species (% of total number of species of taxonomic group in Sonora in parentheses) in different taxonomic groups found in the ecoregions of Sonora, Mexico (see text for description of the ecoregion types).

	Western mainland deserts	High northeastern valleys	Eastern mountains	Subtropical lowlands and foothills	Marine	Island
Amphibia	18 (50)	14 (39)	21 (58)	22 (61)	0 (0)	2 (6)
Anura	17 (52)	13 (39)	19 (58)	22 (67)	0 (0)	2 (6)
Caudata	1 (33)	1 (33)	2 (67)	0 (0)	0 (0)	0 (0)
Reptilia	76 (48)	40 (31)	83 (53)	61 (39)	7 (4)	29 (18)
Crocodylia	0 (0)	0 (0)	0 (0)	0 (0)	1 (100)	0 (0)
Squamata	70 (50)	38 (27)	76 (54)	54 (39)	1 (0.07)	28 (20)
Lacertilia	32 (48)	17 (26)	31 (47)	16 (24)	0 (0)	15 (23)
Serpentes	38 (51)	21 (28)	45 (61)	38 (51)	1 (1)	13 (18)
Testudines	6 (40)	2 (13)	7 (44)	7 (44)	5 (31)	1 (6)
**Total**	**94 (49)**	**54 (28)**	**104 (54)**	**83 (43)**	**7 (4)**	**30 (16)**

**Table 6.** Summary of the numbers of species shared between Sonora and neighboring Mexican states (not including introduced species). The percent of species from Sonora shared by a neighboring state are given in parentheses. Key: – indicates neighboring state has no species in the taxonomic group, thus no value for shared species is provided.

	Sonora	Arizona	Baja California	Sinaloa	Chihuahua	New Mexico
Class Amphibia	36	16 (44)	6 (17)	25 (69)	30 (83)	13 (36)
Order Caudata	3	1 (33)	0 (0)	1 (33)	3 (100)	1 (33)
Ambystomatidae	2	1 (50)	–	1 (50)	2 (100)	1 (50)
Plethodontidae	1	0 (0)	0 (0)	–	1 (100)	0 (0)
Order Anura	33	15 (45)	6 (18)	24 (73)	27 (82)	12 (36)
Bufonidae	12	6 (50)	4 (33)	8 (67)	9 (75)	5 (42)
Craugastoridae	3	1 (33)	–	2 (67)	2 (67)	1 (33)
Eleutherodactylidae	1	–	–	1 (100)	1 (100)	–
Hylidae	6	3 (50)	0 (0)	5 (83)	5 (83)	2 (33)
Leptodactylidae	1	–	–	1 (100)	–	–
Microhylidae	2	0 (0)	–	2 (100)	2 (100)	0 (0)
Ranidae	6	3 (50)	1 (17)	4 (67)	6 (100)	2 (33)
Scaphiopodidae	2	2 (100)	1 (50)	1 (50)	2 (100)	2 (100)
Class Reptilia	158	88 (56)	36 (23)	85 (54)	94 (59)	61 (39)
Order Crocodylia	1	–	–	1 (100)	–	–
Order Testudines	16	4 (25)	5 (31)	12 (75)	6 (38)	2 (12)
Cheloniidae	4	–	4 (100)	4 (100)	–	–
Dermochelyidae	1	–	1 (100)	1 (100)	–	–
Emydidae	4	1 (25)	0 (0)	2 (50)	2 (50)	1 (25)
Geoemydidae	1	–	–	1 (100)	1 (100)	–
Kinosternidae	4	2 (50)	–	2 (50)	2 (50)	1 (25)
Testudinidae	2	1 (50)	–	2 (100)	1 (50)	–
Order Squamata	141	84 (60)	31 (22)	72 (51)	88 (62)	59 (42)
Suborder Lacertilia	66	37 (56)	12 (18)	25 (38)	32 (48)	29 (44)
Anguidae	1	1 (100)	0 (0)	1 (100)	1 (100)	1 (100)
Crotaphytidae	4	3 (75)	1 (25)	–	2 (50)	2 (50)
Dactyloidae	1	–	–	1 (100)	1 (100)	–
Eublepharidae	2	1 (50)	1 (50)	1 (50)	0 (0)	1 (50)
Helodermatidae	2	1 (50)	–	2 (100)	1 (50)	1 (50)
Iguanidae	6	2 (33)	2 (33)	2 (33)	1 (17)	–
Phrynosomatidae	29	20 (69)	6 (21)	12 (41)	18 (62)	17 (59)
Phyllodactylidae	4	–	1 (25)	2 (50)	1 (25)	–
Scincidae	3	2 (67)	0 (0)	2 (67)	3 (100)	2 (67)
Teiidae	12	6 (50)	1 (8)	2 (17)	4 (33)	5 (42)
Xantusidae	2	1 (50)	0 (0)	–	–	–
Suborder Serpentes	75	47 (63)	19 (25)	47 (63)	56 (75)	30 (40)
Boidae	2	1 (50)	1 (50)	1 (50)	1 (50)	–
Colubridae	40	28 (70)	10 (25)	26 (65)	29 (72)	17 (42)
Dipsadidae	9	3 (33)	2 (22)	7 (78)	7 (78)	3 (33)
Elapidae	3	1 (33)	1 (33)	3 (100)	2 (66)	1 (33)
Leptotyphlopidae	1	1 (100)	1 (100)	1 (100)	1 (100)	0 (0)
Natricidae	7	3 (43)	1 (14)	3 (43)	7 (100)	3 (43)
Viperidae	13	10 (77)	3 (23)	6 (46)	9 (69)	6 (46)
Total	194	104 (53)	42 (22)	110 (57)	124 (64)	74 (38)

## Appendix 1

Museum collections included in the CONABIO database examined for records of Sonoran amphibians and reptiles or that house specimens of the first record of a species in Sonora.

**AMNH** Collection of Herpetology, Herpetology Department, American Museum of Natural History

**ANSP** Collection of Herpetology, Herpetology Department, Academy of Natural Sciences of Philadelphia

**ASNHC** Herpetology Collection, Angelo State Natural History Collections, Angelo State University

**ASU** Arizona State University

**NHMUK** Collection of Herpetology, Zoology Department, The Natural History Museum, London, UK

**BYU** Monte L. Bean Life Science Museum, Brigham Young University, Provo, Utah

**CAS** Collection of Herpetology, Herpetology Department, California Academy of Sciences

**CMNH** Collection of Herpetology, Amphibian and Reptile Section, Carnegie Museum of Natural History, Pittsburgh

**CNAR** Colección Nacional de Anfibios y Reptiles, Instituto de Biología UNAM

**CUMV** Amphibian and Reptile Collection, Cornell University Museum of Vertebrates

**ENCB** Colección Herpetológica, Departamento de Zoología, Escuela Nacional de Ciencias Biológicas

**FMNH** Division of Amphibians and Reptiles, Field Museum of Natural History

**FSM-UF** Collection of Herpetology, Florida State Museum, University of Florida

**LACM** Collection of Herpetology, Herpetology Section, Natural History Museum of Los Angeles County

**LEUBIPRO** Laboratorio de Biología UBIPRO

**LSUMZ** Collection of Herpetology, Museum of Zoology, Biological Science Division, Louisiana State University

**MCZ** Collection of Herpetology, Museum of Comparative Zoology, Harvard University Cambridge

**MNHUK** Museum of Natural History, Division of Herpetology, University of Kansas

**MPM** Herpetology, Milwaukee Public Museum

**MVZ** Collection of Herpetology, Museum of Vertebrate Zoology, Division of Biological Sciences, University of California Berkeley

**MZFC-UNAM** Colección Herpetológica, Museo de Zoología “Alfonso L. Herrera”, Facultad de Ciencias UNAM

**PBDB** Paleobiology Database, Paleobiology Database Chordates

**ROM** Department of Herpetology, Royal Ontario Museum, Toronto, Ontario, Canada

**SDNHM** Collection of Herpetology, Herpetology Department, San Diego Natural History Museum

**TCWC** Collection of Herpetology, Texas Cooperative Wildlife Collection, Texas A&M University

**TNHC** Collection of Herpetology, Texas Natural History Collection, University of Texas Austin

**TU** Collection of Herpetology, Biology Department, Tulane University, New Orleans

**UABC** Colección Herpetológica, Universidad Autónoma de Baja California

**UAZ** Amphibians and Reptiles Collections, University of Arizona

**UCM** Collection of Herpetology, University of Colorado Museum

**UIMNH** Collection of Herpetology, University of Illinois Museum of Natural History

**UIUC** Collection of Herpetology, Museum of Natural History, University of Illinois at Urbana-Champaign

**UMMZ** Collection of Herpetology, Museum of Zoology, University of Michigan Ann Arbor

**UMNH** Reptiles and Amphibians Collection, Natural History Museum of Utah

**USNM** Collection of Herpetology, Department of Vertebrate Zoology, National Museum of Natural History, Smithsonian Institution

**UTAMM** Merriam Museum, University of Texas Arlington

**UTEP** Collection of Herpetology, Laboratory of Environmental Biology, Biological Science Department, University of Texas – El Paso
